# Acyclic Identification of Aptamers for Human alpha-Thrombin Using Over-Represented Libraries and Deep Sequencing

**DOI:** 10.1371/journal.pone.0019395

**Published:** 2011-05-19

**Authors:** Gillian V. Kupakuwana, James E. Crill, Mark P. McPike, Philip N. Borer

**Affiliations:** 1 Graduate Program in Structural Biology, Biochemistry and Biophysics, Syracuse University, Syracuse, New York, United States of America; 2 AptaMatrix, Inc., Syracuse, New York, United States of America; 3 Department of Chemistry, Syracuse University, Syracuse, New York, United States of America; The Scripps Research Institute, United States of America

## Abstract

**Background:**

Aptamers are oligonucleotides that bind proteins and other targets with high affinity and selectivity. Twenty years ago elements of natural selection were adapted to *in vitro* selection in order to distinguish aptamers among randomized sequence libraries. The primary bottleneck in traditional aptamer discovery is multiple cycles of *in vitro* evolution.

**Methodology/Principal Findings:**

We show that over-representation of sequences in aptamer libraries and deep sequencing enables acyclic identification of aptamers. We demonstrated this by isolating a known family of aptamers for human α-thrombin. Aptamers were found within a library containing an average of 56,000 copies of each possible randomized 15mer segment. The high affinity sequences were counted many times above the background in 2–6 million reads. Clustering analysis of sequences with more than 10 counts distinguished two sequence motifs with candidates at high abundance. Motif I contained the previously observed consensus 15mer, Thb1 (46,000 counts), and related variants with mostly G/T substitutions; secondary analysis showed that affinity for thrombin correlated with abundance (K_d_ = 12 nM for Thb1). The signal-to-noise ratio for this experiment was roughly 10,000∶1 for Thb1. Motif II was unrelated to Thb1 with the leading candidate (29,000 counts) being a novel aptamer against hexose sugars in the storage and elution buffers for Concanavilin A (K_d_ = 0.5 µM for α-methyl-mannoside); ConA was used to immobilize α-thrombin.

**Conclusions/Significance:**

Over-representation together with deep sequencing can dramatically shorten the discovery process, distinguish aptamers having a wide range of affinity for the target, allow an exhaustive search of the sequence space within a simplified library, reduce the quantity of the target required, eliminate cycling artifacts, and should allow multiplexing of sequencing experiments and targets.

## Introduction

DNA and RNA aptamers [1], [2], [3], [4] have affinities for their targets similar to antibodies [5] and are useful in biosensors [6], [7], [8], [9], diagnostics [5], [10], [11] and therapeutics [12], [13], [14]. Unlike antibodies, aptamers are not limited to binding immunogenic epitopes but have been reported to specifically bind an array of small organic molecules, macromolecules, and cells [3], [4], [9], [15]. Aptamers have also been utilized as *in vivo* tools to detect and influence biological interactions in proteomics and metabolomics research [16], [17]. In contrast to antibodies, aptamers can be prepared by standard solid-phase synthesis at a fraction of the cost for antibodies, have shelf lives of years, and require no animal or cell lines. In addition to their diverse functionality, aptamers are stable over a wide range of pH and temperatures, are not immunogenic and have shown successful protection from degradation by chemical protection [3], [4], [18]. Aptamers for several hundred targets have been described in the open literature [3], [4] over the past 20 years and perhaps a few hundred more have been discovered in unpublished efforts. Far more antibodies have been found in the same time period. Some of this is due to the larger number of scientists who specialize in antibodies compared to those focused on aptamers. However, much of the disparity can be ascribed to the lengthy, cyclic methods in common use for aptamer discovery.

**Figure 1:**
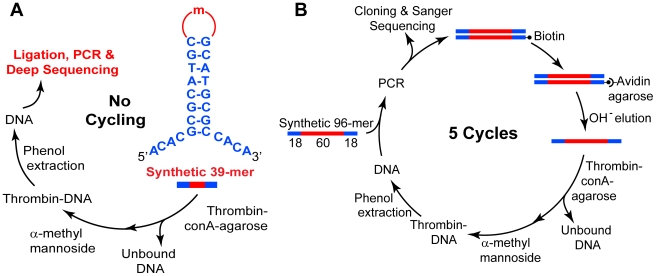
Acyclic approach vs. SELEX for distinguishing aptamers to human α-thrombin. **A**. Starting libraries used here for acyclic selection are structurally constrained and short enough to over-represent each possible library member. **B**. The library used for SELEX was sparsely sampled, where any unique full-length sequence has a very low probability of occurrence (5×10^−23^). There were 34 major tasks in the original work [35], and only 6 in the acyclic protocol used to discover the same thrombin binding aptamer. Figure adapted from Bock *et al.* [35].

In vitro evolution [2], [19], often called SELEX [1], [20], [21], is the standard method for aptamer discovery. SELEX, compared to our acyclic protocol in Figure 1, typically uses five to fifteen cycles of target-partitioning and amplification to enrich aptamer candidates from a pool containing randomized segments of length, m, where m ≥ 30 is typical (Figure 1b).

Simpler and faster methods for aptamer discovery have been sought to shorten the rather complex cycle of enrichment of naïve pools with molecules that have high affinity for their target. Improvements include a robotic SELEX workstation to perform multiple selection cycles [22], [23], [24], reducing tedious sample manipulations. Efficient separation techologies have been applied, including capillary electrophoresis [25], [26], [27], [28], monoLEX [15], which joins a selection step with column fractionation, and a microfluidic device [29]. These methods have been successful in reducing the discovery time from weeks to days. Other methods include photoSELEX, a method that covalently links high affinity binding sequences to their target of interest via a UV radiation-assisted photoreaction [30], The work described in this report [31], [32] and other recent papers [29], [33] have applied deep sequencing to further reduce the cycling requirement in aptamer discovery.

Typical starting libraries for SELEX have oligomers with central randomized regions (30–70 nt) flanked by fixed regions needed for amplification and cloning (overall length often ≥ 90 nt). This is illustrated in Figure 1b for human α-thrombin, where the aptamer core sequence is only 15 bases, as originally deduced by Bock et al. [34]. They used five SELEX cycles, starting with 100 pmol of a 96mer DNA library with a 60 nt randomized region. Most possible sequences are not represented in naïve (unpartitioned) SELEX pools, *e.g.*, in 100 pmol of a pool with m = 60, the probability is only 5×10^−23^ that a particular 60mer segment is present (see Table 1). Thus, virtually all of the 6×10^13^ molecules in the pool occur as single copies. Despite this sparse representation, high affinity molecules that do exist come to dominate the evolving pool under the selective pressure of binding. Repeated motifs identify aptamers in the final pool.

**Table 1:** Representation of sequences in naïve pools.^a^

m^b^	U^c^	R (0.1)^d^	R (25)^d^
**5**	1.0×10^3^	5.9×10^10^	1.5×10^13^
**10**	1.0×10^6^	5.7×10^7^	1.4×10^13^
**15**	1.1×10^9^	56,000	1.4×10^7^
**18**	6.9×10^10^	880	2.2×10^5^
**19**	2.7×10^11^	220	55,000
**20**	1.1×10^12^	55	14,000
**21**	4.4×10^12^	14	3,400
**22**	1.8×10^13^	3.4^e^	860
**23**	7.0×10^13^	0.86	210
**24**	2.8×10^14^	0.21	53
**25**	1.1×10^15^	5.3×10^−2^	13
**26**	4.5×10^15^	1.3×10^−2^	3.3^e^
**30** f	1.2×10^18^	5.2×10^−5^	1.3×10^−2^
**40**	1.2×10^24^	5.0×10^−11^	1.2×10^−8^
**60**	1.2×10^36^	4.5×10^−23^	1.1×10^−20^

a. See Table S1 for a more extensive list and details o calculations.

b. m = length of randomized sequence.

c. U = 4^m^, the number of possible unique sequences.

d. R = the average representation of each unique molecule in a pool, for the indicated (0.1 or 25) nmol of total strands.

e. Beyond diversity limit; some possible sequences are absent.

f. Beyond synthesizer limit for 1 µmol scale synthesis; some sequences are not produced.

It occurred to us that application of deep sequencing to over-represented libraries could distinguish aptamers after a single partitioning step. Such over-represented libraries can be designed within secondary structural motifs that are known to be rich territories for aptamer discovery, as in hairpin loops (Figure 1a). Fairly short randomized regions are required for acyclic identification as over-representation is difficult to achieve for m>25 when the full complement of A, C, G, T(U) occurs at each randomized position. The use of such structured libraries may also avoid the necessity for truncating aptamer candidates to discover minimal core binding sequences.

We chose thrombin as a target to validate acyclic identification, and found the canonical DNA aptamer and lower affinity relatives within a naïve library that substantially over-represents each possible sequence. This library was also adapted to the sequencing platform we used (Illumina) with short read lengths. A natural result of the investigation was that all possible thrombin binding sequences within the sequence space encompassed by the m = 15 library were evaluated [31], [35]. The acyclic protocol reduced the sample manipulations by a factor of six compared to [34], and required only standard methods in molecular biology coupled with deep sequencing that is now widely available.

## Results

### Library design and optimization

We designed a 39mer DNA library with a constant stem to present the m = 15 variable segment in the context of a hairpin loop (Figure 1a). A fixed-sequence stem was chosen to confer reasonable stability in partitioning (T_m_ = 70–75°C over the range of 0.1–0.3M Na^+^ for an unstructured 15mer hairpin loop), while maintaining efficient ligation and PCR. The four-base non-complementary tails shown in the figure were tested to ensure efficient ligation into a sequencing cassette adapted for the Illumina Genome Analyzer platform (GA, data not shown). The sequencer was capable of generating reads up to 36 bases (the read length increased after this work was completed).

Prior to partitioning with a protein target, we tested for bias in the distribution of the four bases in the randomized positions due to the details of library synthesis. A set of m = 6 stem-loops, as in Figure 1a, was “machine-mixed” using what should have been identical molar amounts of each amidite for each randomized loop site; GA-sequencing demonstrated considerable bias as evidenced by the proportion of homo-oligomers, G6:A6:T6:C6 = 0.63: 0.37: 0.0025: 0.0006, which is far from the desired mole fraction of 0.25 for each of these 6mers. This strong bias was corrected in a “hand-mixed” library, where the four phosphoramidites were mixed prior to synthesis and added from one port of the synthesizer (IDT, Coralville, IA). The resultant proportion still showed a slight bias against C-containing sequences, G6:A6:T6:C6 = 0.27: 0.24: 0.29: 0.20, but was gauged to be satisfactory for our purposes.

The possibility that PCR-artifacts might skew the distribution of sequences obtained from Illumina sequencing was also assessed. The data just presented for m = 6 also shows that there is little PCR-bias for or against loop sequences containing homo-oligomer runs of six. Another test for PCR-bias within the context of the loops in Figure 1a was conducted with four different specified m = 15 loop sequences mixed in the molar proportion: 1.00: 0.10: 0.010: 0.0010 with no partitioning against a target. The counts of 3.2 million sequenced clusters were proportional to the dose, 1.00: 0.11: 0.012: 0.0010, accurately representing the input population for these loops over three orders of magnitude. While bias in PCR and deep sequencing is known to occur, these experiments reduced concerns that adapter ligation, PCR, and bridge amplification might introduce a large bias in the sequencing results. However, PCR-artifacts associated with skipping parts of the loop sequence have been detected, as described later.

### Aptamer Selection and Identification

Partitioning conditions used in this study were exactly as described by Bock et al. [34]. Early rounds of SELEX aim to prevent the accidental loss of high affinity aptamer candidates by avoiding competition between candidates for binding sites (typical libraries begin with single copies of any unique full-length sequence; see Tables 1 and S1). Thus, a starting ratio of 60∶1 thrombin:DNA was used by Bock et al., and was used in the current study in order to preserve a direct comparison between SELEX and the acyclic approach introduced here.

High affinity candidates were partitioned, isolated and prepared for sequencing by ligation of Illumina sequencing adapters and PCR-amplification (Figure S1). Following sequencing, the variable regions of output reads were aligned with respect to the invariant stem and tail regions using a Perl script (Table S2). Typically 80–90% of the sequences matched closely enough to the invariant sequences and length of the variable region to merit designation as qualified reads. The base calls for the invariant regions in qualified reads exhibited >95% accuracy for each base position with regard to substitution, deletion, and insertion (Table S3). Output sequences were ranked by the number of times each was counted. The high efficiency of our acyclic procedure is evident in Table 2, which displays the summary statistics for experiment T1. 88% of the total reads passed qualification by the PERL script. Most sequences that are counted only once arise from molecules that have little affinity for the target but are accidentally carried forward in the partitioning step. 82% of the sequences in experiment T1 were counted 1 time only and only 8 out of the 1,728,220 unique sequences were counted more than 500 times. While the total number of counts and the number of sequences in each motif vary between experiments, the trends remain similar (Table S4).

**Table 2:** Summary of sequencing results for experiment T1.

Total reads	2,142,146
Total qualified reads	1,959,748
Unique qualified reads	1,728,220
Count = 1	1,598,788
Count = 2	117,221
Count = 3	10,909
Count = 4−10	1,194
Count = 11−25	43
Count = 26−50	18
Count = 51−100	22
Count = 101−250	13
Count = 251−500	3
Count>500	8

**Figure 2:**
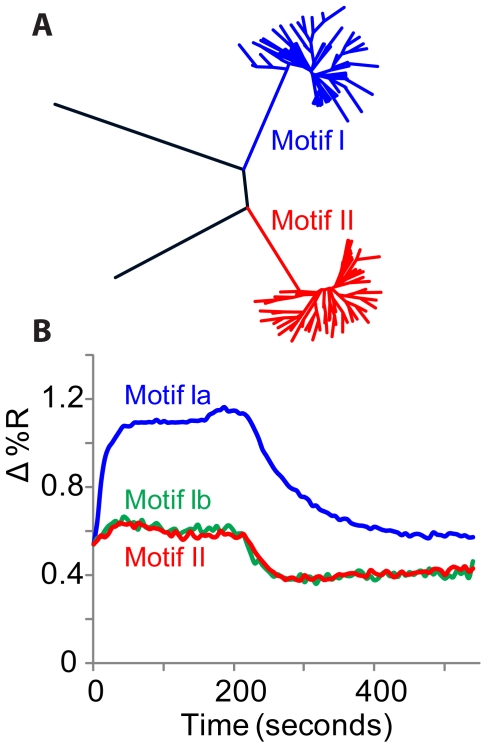
Analysis of selected aptamer candidate sequences. **A**. Motif (phylogeny) tree of sequences with ≥10 counts (108 sequences), in the experiment summarized in Table 2. Motif I at the upper right (blue) binds thrombin, motif II at the lower right (red) binds carbohydrates, while “jump” sequences (black) at the left are PCR-artifacts. **B**. SPR analysis of top contenders from each motif. Thb1 is the top candidate in motif Ia (close relatives of Thb1) and binds strongly to thrombin (upper SPR trace, blue). The top candidate in motif Ib (distant relatives of Thb1, see Table 3) was counted ∼1,000 times less than Thb1 and binds weakly (green trace). Motif II also binds weakly to thrombin (red trace). The SPR signal, Δ%R, is the change in reflectivity of the chip surface in response to analyte.

Sequence alignment within the variable region was made using ClustalX and motif (phylogeny) diagrams were made with Drawtree. Analysis of all sequences from experiment T1 with counts ≥ 10 revealed two major sequence motifs (Figure 2a). Table 3 collects data from this experiment on several members of each motif. Motif I contained the consensus G-quadruplex 15mer from Bock et al. (Thb1), which ranked highest occurring 46,444 times in 4.7×10^6^ qualified reads; this motif also includes variants having mainly G, T substitutions, designated as motif Ia and low abundance variants having many A, C substitutions, designated as motif Ib. Motif II was unrelated to motif I, containing novel carbohydrate binding aptamer sequences related to Carb1 (counted 29,405 times). Three thrombin partitioning and sequencing experiments were conducted, each showing a similar set of sequences from the sequence motifs (see Table S4).

**Table 3:** Aptamer sequences and K_d_ values (in µM).^a^

Rankb	Sequence	Countc	Tbd	Glcd	AMMd
**Motif Ia (top sequence is Thb1)**					
1	**GG**TT**GG**T**G**T**GG**TT**GG**	46,444	0.012	2.5	0.78
3	**GG**TT**GG**T**G**T**GG**TTT **G**	2,451	0.17		
9	**GG**TT**GG**TTT**GG**TT**GG**	419	0.65		
25	**GG**TT**GG**T**GcGG**TT**GG**	96	2.2		
**Motif Ib (more distant Thb1 relatives)**					
51	aGT**G**T**GG**T**c****GG**aa**G**T	53			
54	aT**G**T**GGcG**a**GG**aT**G**a	48			
56	TaT**G**T**GGG**T**G**aaT**Gc**	42			
121	**G**TT**GG**T**G****GcGG**aa**GG**	10			
**Motif II (top sequence is Carb1)**					
2	**Gc**TaT**c**aT**cGc**aa**cG**	29,405		1.4	0.52
4	**Gc**TaT**c**aT**cGc**a**ccG**	1,040			
10	**Gc**T**c**T**c**aT**cGc**aa**cG**	354			
34	**Gc**TaT**c**aT**c**T**c**aa**cG**	80			

a. See Table 2 for counting statistics. Four sequences from each motif are shown: underlined bases in motifs I and II differ from the top candidate; bold fonts and capitals are used to highlight motif similarities.

b. Order of abundance in the deep sequencing results.

c. Counts for indicated sequence.

d. K_d_ values (in µM) measured by SPR for Tb = Thrombin, Glc = glucose, AMM = α-methyl mannoside (accuracy estimated at ±30%). K_d_ values for blank cells were not determined.

### Validation of aptamer candidates

**Figure 3:**
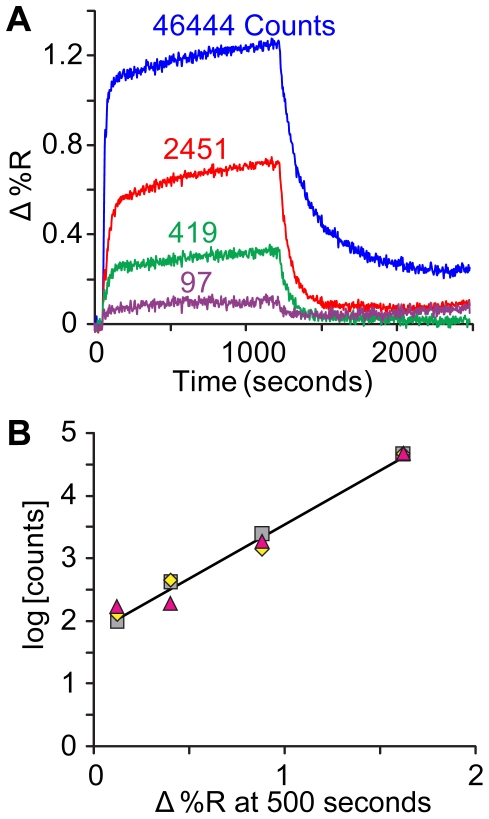
Correlation between counts and binding affinity. **A**. SPR analysis of four sequences from motif Ia having counts over a wide range (see Table 3). **B**. Plot of log[counts] vs. SPR signal for the four sequences in three replicate experiments normalized at the upper right-hand point.

Affinity measurements for thrombin are reported from SPR (Surface Plasmon Resonance imaging) in Table 3 for selected aptamer candidates. SPR traces are shown in Figure 2b for thrombin complexes with the top candidate within each motif immobilized on the SPR chip. Within motif Ia a correlation existed between deep sequencing counts and affinity for thrombin (Figure 3a,b), suggesting that counts may be a useful proxy for affinity within an aptamer family. The estimated K_d_ for the α-thrombin-Thb1 complex (12 nM) was in the range of previous measurements [4]. Motifs Ib and II made weaker complexes with thrombin (Figure 2b).

### Sugar binding aptamer candidate (Carb1)

**Figure 4:**
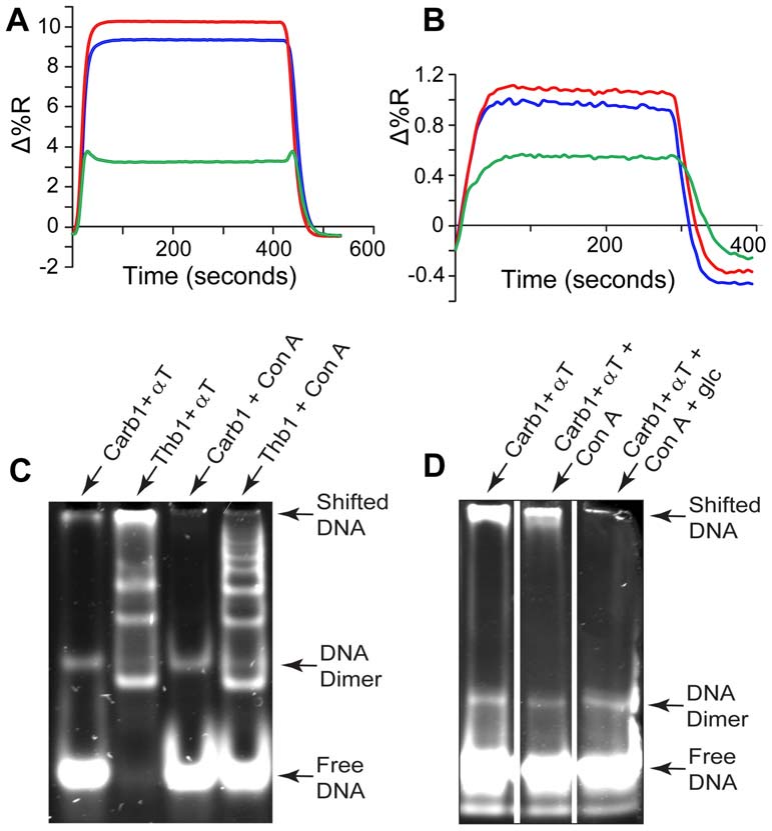
Motif II sequences bind carbohydrate moieties. SPR analysis of (Carb1, red), Thb1 (blue) and an A_15_ control (green) with **A**, α-methyl-mannoside (AMM) and **B**, glucose (glc; note the expanded vertical scale). **C**, **D**, Electrophoretic mobility shift analysis: The affinity of Carb1 for α-thrombin (αT) diminished on addition of Con-A, and disappeared on addition of glucose. DNA hairpins consistently had two bands in EMSAs, the upper (minor) band presumably due to a low concentration of dimerized hairpins.

The weak SPR response to thrombin by the leading sequence from motif II, even though it had the second highest count (29,405), prompted us to investigate the likelihood that the sequence was a ligation artifact or a PCR-champion. Comparative studies of ligation efficiencies and semi-quantitative real-time PCR with Thb1 suggested that Carb1 was not a PCR-champion (Figure S2). A quantitative comparison of ligation efficiency for Thb1 and Carb1 also showed no significant difference (data not shown). However, we noted that the ratio of counts for Carb1/Thb1 increased with increasing concentrations of hexose sugars present in the partitioning step (see Table S4). While Carb1 bound α-thrombin weakly, its SPR-response was high for the two hexose sugars in the storage and elution buffers for Concanavilin-A (ConA) agarose, which was used to immobilize thrombin. This novel sequence bound both α-methyl mannoside (AMM) and glucose (Figure 4); K_d_ ∼500 nM for AMM and ∼1.4 µM for glucose), with the Carb1-AMM affinity ranking in the top third of aptamer-small molecule complexes [4]. Thb1 also has affinity for both sugars consistent with reports that G-rich sequences can be carbohydrate aptamers [36], [37]. Low affinity of Carb1 for thrombin was confirmed by electrophoretic mobility shift assays (Figure 4c,d). The shifted band diminished in the presence of ConA, which competes for the glycosylated residues of thrombin, and disappeared on addition of glucose or AMM. Agarose is composed of galactopyranose units, which might also contribute to the selection of motif II, although negative selection was performed by passing the library through ConA-agarose without thrombin. Future work will distinguish the preference of Carb1 for various sugars.

### Variation in the Thb1 core binding sequence

**Figure 5:**
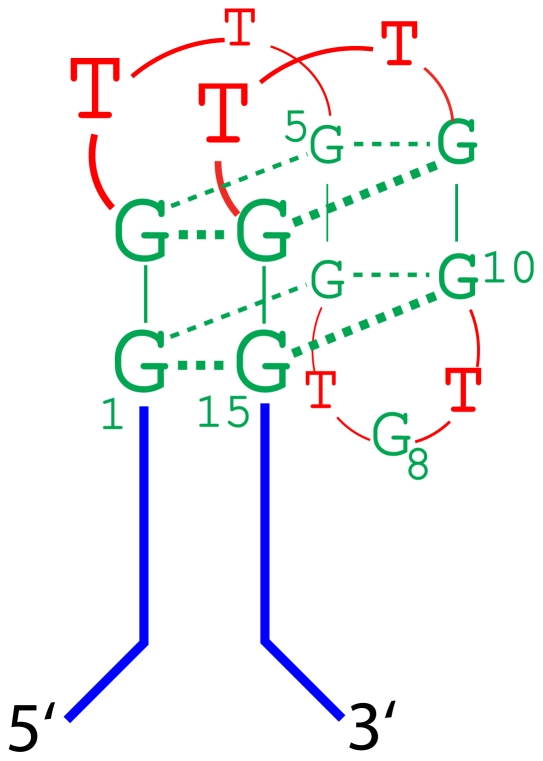
Folding of the canonical thrombin binding sequence. In Thb1 the 15mer hairpin loop folds into the G-quadruplex structure on the stem and tails shown in Figure 1.

The consensus thrombin binding aptamer (Thb1) discovered by Bock *et al.* forms two stacked G-quartets connected by three loops; the minimal form that retains high activity has fifteen residues [34], [38], [39] (Figure 5). We aligned the first 54,140 counts of motif I (Thb1 motif) composed of 108 sequences and determined the frequency of each of base in the 15 variable positions (Table S5). Work by others has interrogated the sequence space for a thrombin aptamer using high density microarrays [40], and has produced similar rankings of affinity for thrombin.

### Functional assay

**Figure 6:**
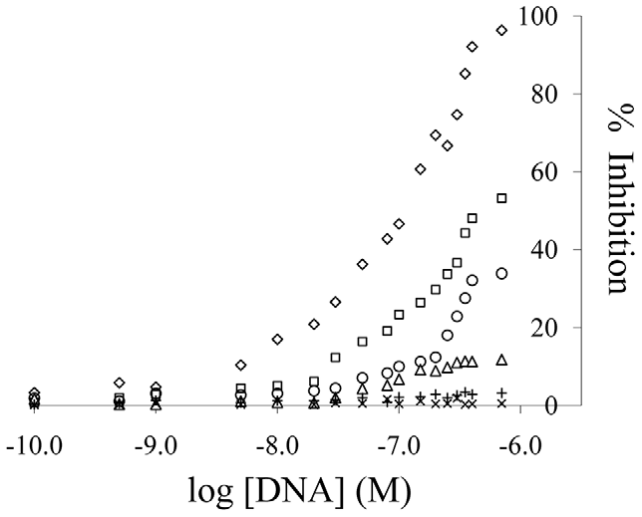
Effect of selected motif I aptamers on clotting activity of α-thrombin. Thrombin activity was measured in the presence of varying concentrations of selected sequences with the corresponding counts shown in the legend (the sequences are listed in Tables 3 and S4). Motif II sequences had no effect on the clotting times.

As expected Thb1 strongly inhibited thrombin's enzymatic activity [34], while variants had reduced performance in the order of their counts (Figure 6; see Tables 3 and S4 for sequences of the 6 oligonucleotides used in the clotting assay). Structural data has established that the Thb1 G-quadruplex binds α-thrombin's anion-binding exosite I, effectively inhibiting its protease activity [38], [39], [41]. Carb1 and its related motif II sequences had no observable effect on thrombin's activity suggesting that its (weaker) binding site is elsewhere, perhaps on the glycosylated periphery of the protein.

### Estimation of the noise threshold

Clustal was also used to estimate the contribution of background noise to the sequence counts. Figure S3 shows tree diagrams at thresholds of count = 10 (used for Fig. 2a) down to count = 4 (at a count threshold of four, there were more than 1000 sequences, the upper limit for ClustalX, so not all sequences were included). As the threshold was reduced, more and more sequences were included in Motifs I and II, and a progressively larger number of sequences were uncorrelated to either Motif I or II. At a threshold of five, these uncorrelated “noise” sequences were roughly equal in population to the “signal” sequences in Motifs I and II. Going from a threshold of five to four, new signal sequences appeared, but the noise sequences became the overwhelming majority.

## Discussion

### Representation

There is the potential for confusion with regard to the word, sampling, which is often used to describe the occurrence of candidate sequences in an aptamer library, with the degree of sampling afforded by deep sequencing. We prefer to use *sampling* for the latter, *i.e.*, to take a sample for sequencing. The term used here to describe the occurrence of unique sequences within an aptamer library is the *average representation*, R. The number of possible *unique sequences*, U, in a pool with m randomized nucleotides is 4^m^ when four nucleotides occur at each randomized position in equal population.

For the 6×10^13^ molecules in 100 pmol of a library with m = 15, U is 1.1 billion unique molecules; R is 56,000 for each of these, making this an *over-represented* library (see Tables 1 and S1). For a library with m = 22 and R = 3.4, R is the average taken over a distribution of discrete objects so the largest number of sequences are present in three copies and nearly as many occur in four copies. Such a random distribution of 18 trillion unique molecules in a total of 60 trillion will have some sequences present in two and five copies, as well as other multiples. Poisson statistics predict that there will be some sequences present in zero copies–absent from the distribution. We refer to m = 22 as being beyond the *diversity limit* for 100 pmol of library; full representation of all sequences is unlikely. By contrast, Poisson statistics predict for m = 21 (R = 14) that the overwhelming majority of possible sequences are present. As noted in the Introduction, R = 5×10^−23^ when m = 60; thus, the original pool of Bock et al. [34] was very *sparsely represented*. In such a pool (whenever R<<1) it is extremely unlikely for any sequence to occur in more than one copy.

At a pool size of 25 nmol, m = 26 is beyond the diversity limit (last column of Table 1). It is common to perform library synthesis on the 1,000 nmol scale so acyclic aptamer discovery with m = 25 should be practical if a sufficient amount of the target is available. However, the *synthesizer limit* is passed at m = 30 for a 1,000 nmol synthesis. That is, U = 1.2×10^18^ and fewer molecules will be produced from synthesis at this scale (<6×10^17^). While it is possible to increase the synthesis scale, it may become prohibitively expensive to supply enough target for acyclic selection and identification.

### Sampling

A primary advantage of using deep sequencing in aptamer discovery is that many more sequences are sampled in the partitioned pool than when Sanger sequencing is utilized. We define the *Sampling Ratio*, SR, as the ratio of total qualified reads to the total number of sequences in the pool. This is the fraction that was physically sampled; in the work presented here SR is taken as the fraction of the partitioned pool that was sampled.

Bock et al. [34] reported 0.01% recovery of DNA from the 100 pmol applied to thrombin immobilized on ConA-agarose beads in the first cycle of SELEX (determined using ^32^P-labeled DNA). Thus, about 6×10^9^ sequences survived partitioning from the initial pool of 6×10^13^. As described above, the overwhelming majority of m = 60 sequences in the initial pool were present as single copies. If Bock et al. had sequenced 32 clones following cycle 1 (as they did after cycle 5) SR would have been 32/6×10^9^ = 5×10^−9^, sampling one molecule per 200 million.

By contrast, the experiments reported in this paper have a much higher value for SR. Assuming that partitioning has the same efficiency as for Bock et al., and for 2 million qualified GA reads for experiment T1 (Table 2), SR = 2×10^6^/6×10^9^ = 3×10^−4^; about 1 molecule was sampled per 3,000 recovered after partitioning.

### Signal to Noise

The counts for authentic aptamers are regarded as the *signal* in our experiment. We have shown that the signal is strongly correlated with binding affinity (Figure 3). If the partitioning step is efficient, the signal for Thb1 should be proportional to the number of Thb1 sequences in the naïve pool. Relatives of Thb1 will have signals reduced by their less efficient retention by the Thb protein in partitioning. The same should hold for Carb1 and its relatives. On the other hand, random *noise* should arise primarily from sequences that are accidentally carried forward in the partitioning step. Most signal sequences will be correlated to Thb1 or Carb1, while noise sequences should be uncorrelated to Thb1, Carb1, and themselves. The noise threshold is due to multiples of non-binding sequences that must be expected for molecules that are sampled randomly from the partitioned pool. As discussed under Sampling, above, there should be about 6×10^9^ total sequences in this pool, not all unique because U = 1.1×10^9^ (Table 1). This distribution is skewed toward sequences having some affinity for the targets. We will show next that the skewness is small-only ∼2% of the sample contains signal sequences. At SR = 3×10^−4^, the 2×10^6^ qualified reads must contain some multiples of noise sequences. (In this analysis, both signal and noise sequences are assumed to be amplified equally by PCR prior to clustering on the GA flow cell. Although PCR may be biased against particular sequences or structural motifs, it is unlikely to strongly alter the distribution of millions of non-binding noise sequences that pass through a selection process by random chance. Neither is it likely to distort the distribution of signal sequences for the Thb1 family, as the counts have been shown to correlate with affinity for thrombin-see Figure 3.)

It is possible to make an empirical estimate of the noise threshold from the Clustal analysis. Most sequences with 9, 8, and 7 counts in experiment T1 are included within the Thb or Carb motif (Figure S3). More outliers become evident as the count is reduced. At a count of five, roughly half of the sequences are no longer classified within the Thb or Carb families, yet new signal sequences continue to accumulate. Uncorrelated sequences dominate at four counts and fewer. Thus, a count of five represents an upper bound on the noise threshold, and four counts is a lower bound.

We conclude that the signal/noise ratio in the thrombin-selection experiment, T1, was very high, specifically, S/N ≅ 10,000 (46,444 divided by 4 or 5). There were 89,761 total counts for all unique sequences with five counts or more; this group, constituting 1.9% of all of the qualified reads, is clearly dominated by signal sequences. It is likely that some weaker-binding signal sequences occur at 4 counts and less, and that there are sequences that are unrelated to either Thb1 or Carb1 that have low, but non-zero affinity. Thus, there may be more than 100,000 total signal sequences in the data set. Most of the unique sequences (1.6 million), were counted only once and most appear to be uncorrelated to Thb1 or Carb1. At 0.01% recovery [34], as discussed above, 6×10^9^ sequences would have survived partitioning. If 100,000 signal sequences were present among the GA counts, correcting for SR gives 3×10^8^ signal sequences after partitioning; this still leaves 5.7×10^9^ uncorrelated sequences out of 6×10^9^ total. It is not surprising that the majority of unique reads occur one time only.

### Over-representation limit

It is interesting to estimate the limit in diversity beyond which aptamers can no longer be distinguished by simple counting. We have shown here that acyclic identification is robust for 100 pmol of over-represented libraries with m = 15 (R = 56,000). Due to Poisson noise, the S/N must tend toward one before R is reduced to one. Distinguishing aptamers by counting after acyclic identification cannot succeed when S/N = 1, and must instead rely on motif recognition and/or cyclic enrichment. Preliminary experiments where oligonucleotides were spiked at known concentrations suggested that acyclic identification can be achieved at m = 18 [42] and perhaps longer (R = 880 at m = 18 and 220 at m = 19 see Table 1). It should also be possible to find aptamers in larger pools, e.g., 25 nmol, where m = 22 to 23 have R = 860 and 210 (Table 1). Using these pools should reduce the signal 50 to 300-fold for an aptamer requiring the full diversity of m sites, giving S/N ∼40 if the experiment scales according to our thrombin results. The signal may increase for increasing values of m for aptamers that do not require the full diversity of m sites. For example, when m = 16, there are eight ways to fit the canonical 15mer Thb1 into a 16-base randomized region (four with any base at the 5′-side and four at the 3′-side), and four ways to increase each of the three loops by one base (see Figure 5). Thus, there should be twenty new ways to represent high affinity versions of Thb1 at m = 16, with only a four-fold reduction in all unique sequences in going from m = 15 to 16. This corresponds to a five-fold increase in high-affinity signal, and does not begin to consider all of the new lower affinity versions corresponding to low-count sequences in Figure S3.There is also reason to expect a lower noise threshold from more diverse libraries (m>18). That is because the Poisson distribution predicts that fewer multiples of sequences will occur in these larger libraries, so fewer multiples will be randomly carried forward by partitioning. Therefore, the background could be lower than the estimate of four to five counts determined above. Experiments in progress aim to define practical over-representation limits for acyclic identification.

### Variants of Thb1

The sequencing results were used to explore variability within the Thb motif (see Table S5). The first 6 bases, GGTTGG (Figure 5), were >99% conserved. The largest variability occurred at G8, G10, G14 and G15. The G8 to T8 substitution caused a huge decrease in counts, so the canonical Thb1 is clearly optimal among 15mers. Variability at G14 and/or G15 may be compensated by using the 3′-neighboring stem base (also a G) to complete the G-quartet structure. This would come at the expense of one base-pair in the stem.

Other high-affinity DNA aptamers are known for thrombin that involve quadruplexes. These aptamers adhere to the same general motif outlined in Figure 5; most have longer loops, which are not encompassed within the present libraries.

### Jump sequences

There are systematic artifacts resulting from deletion of a portion of the library, probably due to PCR. Figure S4 shows the most abundant artifact sequence that was a result of the deletion of parts of the library construct for the DNA m = 15 experiment. These jumps escape filtering by PERL scripts as adapter 2 has short stretches nearly identical to the authentic 3′-fixed region (see Figure S4). Perl scripts discriminate against PCR-artifacts, which are a small proportion of the 2–5 million qualified reads. Other PCR-artifacts may exist that do not fit this pattern, so examination of sequence motifs within candidates and assaying for affinity and function are important.

### Comparison with SELEX

The acronym, SELEX, is derived from the term, Systematic Evolution of Ligands by Exponential enrichment. Prominent is the word, evolution. We have described an acyclic procedure for distinguishing DNA sequences with high affinity for proteins or small molecules that does not rely on cyclic evolution.

The variable region within a library of 10^14^ molecules with a large value of m will contain all possible sub-sequences below a certain threshold length (∼22 nt for 100 pmol of library-see Table 1). This has been used to argue that complete representation is unnecessary [19]. However, when each sequence is present only once, as in sparse representation, cyclic enrichment is mandatory before an aptamer can be distinguished by counting. It would be difficult to find a single copy of a high-affinity sequence among the billions of sequences that are accidentally carried forward through the partitioning step.

By contrast, acyclic selection and identification allows the distinction of aptamers simply by counting. We have described how this approach easily located a known thrombin aptamer and lower-affinity variants. In other work, our lab has shown that this approach is compatible with RNA libraries and RNA/DNA chimeric libraries (Chen, L, JEC, MPM, and PNB, unpublished).

It can be difficult to distinguish the key binding elements in long aptamers because contact sites are dispersed in a folded structure that is not known in advance. This may require extensive post-SELEX optimization [20], [43], [44], [45]. Furthermore, diversity in aptamers with long variable regions is often consumed in presenting the combining domain in a favorable context. This suggests that libraries with pre-defined secondary structures can simplify the process of determining the active binding form. It is easy to multiplex such libraries using deep sequencing, either before the partitioning step or in the sequencing step. Winning sequences can be distinguished by matching against the library templates, which have fixed bases and randomized regions of defined length.

There are advantages in combining results from sparse representation and over-representation. For instance, ordinary SELEX can guide construction of over-represented libraries to refine aptamer candidates by acyclic identification. A related item is that SELEX has found aptamers in long randomized regions; such aptamers may allow more widely dispersed contacts with proteins that could be important in some applications. Such long randomized regions are not amenable to acyclic identification as described in this paper. Techniques inspired by SELEX [15], [46], [47] that focus on highly efficient separations can be coupled to deep sequencing in either over-represented or sparsely-represented libraries. Further, over-represented and sparsely represented libraries can be mixed prior to partitioning. Acyclic identification is also compatible with many modified RNA nucleotides that are refractory to enzymatic replication of an evolving pool, but can be copied into DNA for sequencing, e.g., by reverse transcription.

Acyclic identification should afford new opportunities for multiplexing targets. In the absence of cyclic evolution, aptamers with high affinity do not compete against those with moderate or low affinity. Thus, it should be possible to mix multiple targets prior to partitioning against a library, and the aptamers matched to their individual targets in secondary screens. Multiplexing the targets was demonstrated here in the co-isolation of aptamers for α-thrombin and carbohydrates.

We aim to further refine our approach to make empirical determinations of (i) the minimal degree of over-representation, (ii) the effect of varying the molar ratio of target:pool, and (iii) the practicality of motif recognition in acyclic identification. An important part of future work will be to construct libraries that incorporate diversity in productive regions of the space of possible sequences, while maintaining over-representation. This will build upon the success of SELEX over the years in identifying structural motifs that are fertile ground for aptamer discovery. Acyclic identification should help aptamers to reach their full potential in sensing and therapeutic applications.

### Summary

Our current study describes an efficient method to discover aptamers. This approach (i) simplifies and shortens the discovery process, reducing the prospect that human error will compromise the results, (ii) exhaustively searches the space of sequences within a library of pre-defined secondary structures, (iii) may eliminate or reduce experiments to truncate long aptamer sequences to find the core binding domain, (iv) reduces the quantity of the target required, and (v) eliminates cycling artifacts. In addition, acyclic identification should allow multiplexing of targets, and will be compatible with many modified RNA/DNA nucleotides if they can be copied into ordinary DNA by PCR or reverse transcription. The latter is an aspect that can become crucial in applications of aptamers as therapeutics. The principal expense of this approach is the cost of deep sequencing, which is more than offset by the reduction in the amount of target and labor needed to conduct five rounds or more of cyclic evolution. The sequencing cost can be reduced by multiplexing different selection experiments, as is routine for deep sequencing platforms. As sequencers take leaps into billions and trillions of reads, acyclic identification will become even more attractive.

We have also made an exhaustive survey of the space of randomized 15mers applied to their affinity for thrombin and carbohydrates. The canonical thrombin aptamer was distinguished at a signal:noise ratio of ∼10,000∶1. More than 1,000 aptamer candidates were found with counts ≥4, and sequences similar to the canonical thrombin aptamers. Counts for thrombin aptamers correlated with affinity over at least a factor of 200 in K_d_. The primary carbohydrate binding sequence has K_d_ = 500 nM for α-methyl mannoside. This affinity ranks it in the top third of aptamers discovered to date that bind small molecules.

## Methods

### Thrombin analysis by MALDI TOF mass spectrometry

Human α-thrombin was purchased from Haematologic technologies (Essex Junction, VT) in 50% glycerol stocks. Before spotting it on the MALDI plate, the thrombin was dialyzed in 1 liter of PBS buffer twice at 4°C over 24 hours. MALDI TOF mass spectrometry was performed on a Bruker AutoFlex mass spectrometer operated in reflectron mode. Final spectra were the average of 50 shots/position at 10 different positions (Figure S5). The protein was consistently >90% pure with low concentrations of self-cleavage products.

### Aptamer selection

Partitioning, elution of thrombin-DNA complexes, and DNA extraction exactly followed Bock et al. [34]. The m = 15 DNA library was purchased from IDT (Coralville, IA) hand mixed to provide nearly equimolar amounts of the four bases at each degenerate position. 100 pmol of the m = 15 library in 1 mL of partitioning buffer (20 mM Tris-HCl, 140 mM NaCl, 5 mM KCl, 1 mM CaCl2 and 1 mM MgCl2 at pH 7.4) was subjected to negative selection in a 1 mL slurry of pre-equilibrated concanavilin-A beads (Pierce Biotechnology), recovering ∼93% of the DNA. The DNA was then applied to 6 nmol of human α-thrombin (Haematologic Technologies) immobilized on 1 mL Con-A beads equilibrated in partitioning buffer. The column was washed with partitioning buffer, and complexes were eluted with 0.1 M AMM. DNA was recovered by phenol extraction and ethanol precipitation. It is important to check the DNA recovery after negative selection on Con-A beads as some lots of beads were observed to bind more than 90% of the DNA. Even with high recovery after negative selection, some bead lots also exhibited lower recovery of Thb1 and related sequences. This lot-to-lot inconsistency was found with beads from GE Life Sciences as well as those from Pierce.

### Preparation for deep sequencing

Refer to Figure S1 for an overview of preparation for sequencing, and the end of this section for the list of DNA sequences used.

The 3′ strands of the Illumina GA adapters were modified to include sequences complementary to the invariant regions of the m = 15 library. Adapters and complementary splints were added at 50 µM to the partitioned library, heated for 3 min at 90°C, ligated with T4 DNA ligase (New England Biolabs) at 25°C for 30 min, and the reaction terminated by heat denaturing the enzyme at 70°C for 3 min. The reaction was cooled to room temperature, the ligated library (which has two 45 bp double-stranded segments, see Figure S1) was extracted using a QIAquick PCR-purification kit, purified by 2% agarose gel electrophoresis, and stained with ethidium bromide (Figure S6a); visualization of a DNA ladder and the excess adapters helps to choose the region from which to excise the ∼120 nt ligation product from the gel (Figure S6a). The DNA was extracted using a QIAGEN MiniElute Gel Extraction Kit. The library was then PCR-amplified using *Pfu Turbo* DNA polymerase (Stratagene) to extend the flanking regions for clustering on an Illumina flowcell. PCR-conditions were: (a) 2 min at 94°C, (b) 18 cycles of (1 min at 94°C, 1 min at 61°C, and 1 min at 72°C), (c) 10 min at 72°C. The PCR-product was purified using the QIAquick PCR-purification kit and size checked on a 2% agarose gel (Figure S6b) before sequencing by the Illumina GA.

### Sequencing Data Analysis

A PERL script was used to identify sequence strings that closely matched the 5′- and 3′-fixed regions flanking the degenerate bases (F5-m-F3). Table S2 specifies the match regions within F5 and F3, which are adjacent to the central region of m bases. The match criteria were used to generate a file of Qualified reads for sequences with the desired length of central m-bases. An Nmer count file was generated to give the number and rank for each unique sequence.

### Sequence Alignments

All sequences with a count ≥10 were aligned using ClustalX, and plotted using DRAWTREE.

### SPR analysis

Aptamer affinities were measured using a GWC SPRimager®II (GWC Technologies, Inc.) and 16 and 25 SpotReady™ chips at 25°C. SPR data was acquired with V++ imaging software and analyzed in Microsoft Excel. Functionalizing a chip involved its immersion into a 1 mM solution of 8-amino-octanethiol (Dojindo Molecular Technologies, Inc.) in absolute ethanol at room temperature overnight. Rinsing was done with absolute ethanol and drying under nitrogen and incubation in 1 mM 4-(N-maleimidomethyl) cyclohexane-1-carboxylic 3-sulfo-n-hydroxysuccinimide ester (SSMCC) (Pierce Biotechnology) for an hour. Reduced 3′-thiolated oligonucleotides (2 mM) were then spotted in 5 replicates per sequence onto the SSMCC treated chip and allowed to react overnight, then washed with nuclease-free water and dried under nitrogen. The chip was blocked overnight with 4 mM mPEG-thiol (MW 1000) (Nanocs) to cap unreacted SSMCC. Once mounted on the instrument, the chip was blocked with 500 nM BSA (Fisher Scientific), washed with 0.02% Tween-20 in partitioning buffer, and partitioning buffer alone. 50 nM α-thrombin was pumped into the flowcell at 1 ml/min for 10 min after which partitioning buffer was used to wash the chip; the average of background traces (buffer only) was subtracted. Rates of complex formation, k_1_, and dissociation, k_−1_, were estimated by single-exponential fits to the increase in reflectivity upon pumping the analyte over the chip surface, and to the decrease upon pumping buffer; the dissociation equilibrium constant, K_d_ = k_−1_/k_1_.

### Electrophoretic mobility shift assays

Aptamer candidates were heated at 95°C for 3 min and snap cooled on ice for 10 min before pre-incubation for 30 minutes with respective components as explained in each corresponding gel legend, in selection buffer. Rapid cooling was used for these hairpin sequences to limit dimerization involving the self-complementary stems. Samples were analyzed on native polyacrylamide gels (14% (w/v)) in 1X Tris-glycine running buffer at 100 V for 1 hour at 4°C. Immediately after electrophoresis, gels were stained with SYBR gold nucleic acid stain (Invitrogen) for 1 hour, imaged and subsequently stained with Coomassie Brilliant Blue protein stain.

### Semi-quantitative real-time PCR

12 PCR-reactions per aptamer candidate (Thb1 and Carb1) were prepared with equal amounts of starting template DNA and PCR-cocktail reagents. PCR-cycling conditions were as described for the preparation for deep sequencing above but were conducted for 30 cycles instead of 18. Two tubes per sequence were removed at cycle 10, 14, 18, 22, 26 and 30 and the extent of amplification compared by gel electrophoresis and Nanodrop DNA concentration readings.

### Thrombin functional assay

Clotting times were measured in duplicate using a mechanical fibrometer, Oatoclot 2 (Helena Laboratories). Normal human plasma and varying concentrations of DNA aptamer candidates (0.1 nM–700 nM) were incubated for 4 min at 37°C before adding α-thrombin diluted in selection buffer to a final thrombin concentration of 7.5 nM. The extent of thrombin inhibition was calculated from a standard curve generated by measuring the plasma clotting time versus thrombin concentration.

### DNA sequences

All sequences are listed in 5′ to 3′ direction.

Note: Adapter 1 & 2 complements possess overhangs complementary to the constant stem and tail regions of the library from each direction. The forward PCR-primer also introduced a 5′-overhang complementary to an oligomer planted on the Illumina flowcell to anneal the amplified library for sequencing.

*m = 15 DNA library*

Phos/ACACGCGCATGC-m15-GCATGCGCCACA

*Adapter 1/Sequencing Primer*

ACACTCTTTCCCTACACGACGCTCTTCCGATCT

*Adapter 1 complement*

GCATGCGCGTGTAGATCGGAAGAGCGTCGTGTAGGGAAAGAGTGT

*Adapter 2*

Phos/GATCGGAAGAGCTCGTATGCCGTCTTCTGCTTG

*Adapter 2 complement*

CAAGCAGAAGACGGCATACGAGCTCTTCCGATCTGTGGCGCATGC

*PCR-forward primer*

AATGATACGGCGACCACCGAGATCTACACTCTTTCCCTACACGACGCTCTTCCGATCT

*PCR-reverse primer*

CAAGCAGAAGACGGCATACGAGCTCTTCCGATCT

## Supporting Information

Figure S1 **Detailed experimental outline.** **A**. Alpha thrombin was immobilized on concanavalin-A-agarose beads; a library of DNA hairpin loops was applied after negative selection against conA. After several wash steps, high affinity binding sequences were co-eluted with the alpha thrombin. The high affinity binding sequences were extracted by phenol and chloroform extraction and concentrated by ethanol precipitation. **B**. The 15mer library used against alpha thrombin was a hairpin library with the 15mer degenerate library region indicated as region m in red. **C**. After extraction the high affinity binding sequences had adapter constructs ligated as required by the Illumina sequencing platform. Splint strands ensured proper ligation. **D–F**. The ligated, partitioned library was PCR-amplified to introduce a 5′ overhang that annealed the sequences to the complement immobilized on the Illumina flow cell. This was followed by bridge amplification on an Illumina cluster station prior to the sequencing by synthesis process. **G**. The first one or two base reads and reads after base 36 are less accurately determined than the rest; all experiments generated 2 to 5 million, 36mer reads.
(DOCX)

Figure S2 **Investigation of potential amplification bias in aptamer candidates by semi-quantitative real time PCR.** **A**, **B** Analysis of PCR-amplification rates by gel electrophoresis and absorbance measurements at 260 nm. In panel **A** are six pairs of PCR time points for Thb1 (motif I) and Carb1 (motif II), respectively, in a 2% agarose gel (cycle numbers: 10, 14, 18, 22, 26, 30) and in **B**, are the 260 nm readings of a duplicate experiment.
(DOCX)

Figure S3 **Background determination in thrombin aptamer identification.** Following deep sequencing, the occurrence of each sequence was determined and ranked. High occurrence sequences were aligned to determine conserved motifs. The panels show phylogenetic trees generated from sequences counted 10 times or higher, 9+, 8+, 7+, 6+, 5+ and about one thousand of 4+, respectively. Replicate experiments consistently had large numbers of uncorrelated sequences, starting at a count of 4 to 6.
(DOCX)

Figure S4 **Systematic artifacts in surviving sequences.** The full adapter-ligated construct prior to PCR-amplification is shown in **A**. Highlighted in yellow are the stem and tails of the hairpin library designed for the 15mer thrombin experiment, while highlighted in green, the letters, N, signify the 15mer library loop region. The red bases are the flanking regions used by the Perl script to find qualifying reads. In the case of the most abundant systematic artifact, the Perl script recognized the 5′ flanking region but substituted the second flanking region with the adapter sequence underlined above, thereby presenting the first 15 bases of adapter 2 as the library region. This could only occur with the jump shown in dotted line and from the sequence shown in **B**.
(DOCX)

Figure S5 **Thrombin analysis by MALDI TOF mass spectrometry.** Purity of α-thrombin was verified by MALDI TOF mass spectrometry prior to use in selection experiments and was consistently >90% pure with minimal degradation.
(DOCX)

Figure S6 **Sample preparation for high throughput sequencing after selection.** **A.** Confirmation of the ∼120 base pair (bp) ligation product on a 2% agarose gel. The ligation product was excised from the gel, purified and PCR-amplified. **B.** Size confirmation of the final selected, ligated and PCR-amplified pool prior to DNA sequencing. In the first lane of both pictures is a 50 bp DNA ladder. In lane 2 of **A** is the ligation product before excision and clean up. In lanes 2 and 3 of **B** are a negative PCR-control and the PCR-product respectively.
(DOCX)

Table S1 **Representation of sequences in naïve pools.**
(DOCX)

Table S2 **Files generated by Perl script.**
(DOCX)

Table S3 **Base calling statistics for the fixed-sequences flanking the m = 15 library region.**
(DOCX)

Table S4 **Results from three replicate selection and identification experiments for** α**-thrombin with increasing hexose sugar content.**
(DOCX)

Table S5 **Statistics for the sequence space encompassing the Thb1 motif.**
(DOCX)
