# Supplementary material for: Acyclic Identification of Aptamers for Human alpha-Thrombin Using Over-Represented Libraries and Deep Sequencing
Source: PLoS One. 2011 May 19;6(5):e19395. doi: 10.1371/journal.pone.0019395 (PMC3098231; doi:10.1371/journal.pone.0019395)
Supplement: Figure S1 — Detailed experimental outline. A. Alpha thrombin was immobilized on concanavalin-A-agarose beads; a library of DNA hairpin loops was applied after negative selection against conA. After several wash steps, high affinity binding sequences were co-eluted with the alpha thrombin. The high affinity binding sequences were extracted by phenol and chloroform extraction and concentrated by ethanol precipitation. B. The 15mer library used against alpha thrombin was a hairpin library with the 15mer degenerate library region indicated as region m in red. C. After extraction the high affinity binding sequences had adapter constructs ligated as required by the Illumina sequencing platform. Splint strands ensured proper ligation. D–F. The ligated, partitioned library was PCR-amplified to introduce a 5′ overhang that annealed the sequences to the complement immobilized on the Illumina flow cell. This was followed by bridge amplification on an Illumina cluster station prior to the sequencing by synthesis process. G. The first one or two base reads and reads after base 36 are less accurately determined than the rest; all experiments generated 2 to 5 million, 36mer reads. (DOCX) [file pone.0019395.s001.docx]

**
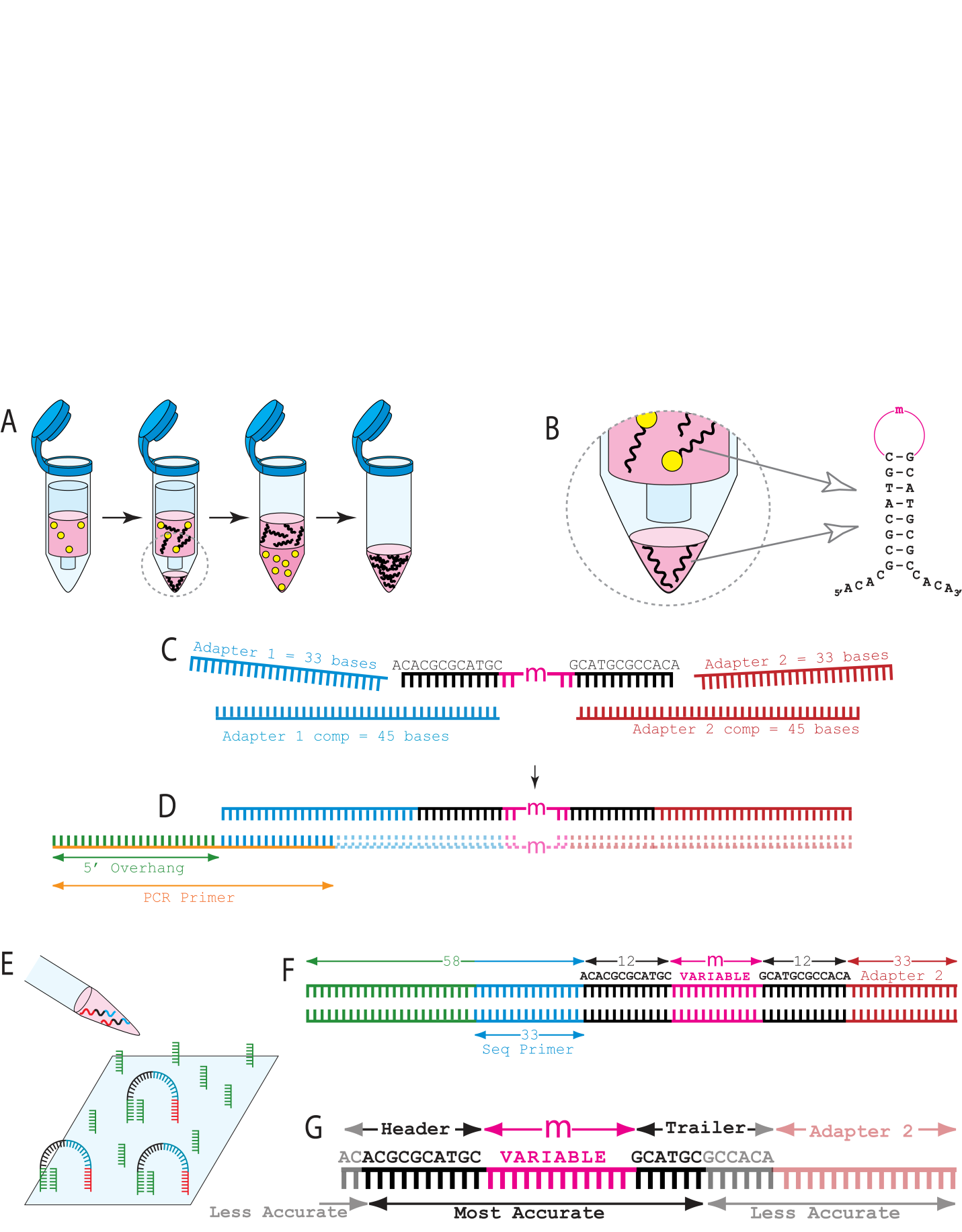
**

**Figure S1. Detailed experimental outline.** **A.** Alpha thrombin was immobilized on concanavalin-A-agarose beads; a library of DNA hairpin loops was applied after negative selection against conA. After several wash steps, high affinity binding sequences were co-eluted with the alpha thrombin. The high affinity binding sequences were extracted by phenol and chloroform extraction and concentrated by ethanol precipitation. **B.** The 15mer library used against alpha thrombin was a hairpin library with the 15mer degenerate library region indicated as region m in red. **C.** After extraction the high affinity binding sequences had adapter constructs ligated as required by the Illumina sequencing platform. Splint strands ensured proper ligation. **D-F.** The ligated, partitioned library was PCR-amplified to introduce a 5' overhang that annealed the sequences to the complement immobilized on the Illumina flow cell. This was followed by bridge amplification on an Illumina cluster station prior to the sequencing by synthesis process. **G.** The first one or two base reads and reads after base 36 are less accurately determined than the rest; all experiments generated 2 to 5 million, 36mer reads.
