# Supplementary material for: Acyclic Identification of Aptamers for Human alpha-Thrombin Using Over-Represented Libraries and Deep Sequencing
Source: PLoS One. 2011 May 19;6(5):e19395. doi: 10.1371/journal.pone.0019395 (PMC3098231; doi:10.1371/journal.pone.0019395)
Supplement: Figure S2 — Investigation of potential amplification bias in aptamer candidates by semi-quantitative real time PCR. A, B Analysis of PCR-amplification rates by gel electrophoresis and absorbance measurements at 260 nm. In panel A are six pairs of PCR time points for Thb1 (motif I) and Carb1 (motif II), respectively, in a 2% agarose gel (cycle numbers: 10, 14, 18, 22, 26, 30) and in B, are the 260 nm readings of a duplicate experiment. (DOCX) [file pone.0019395.s002.docx]

**
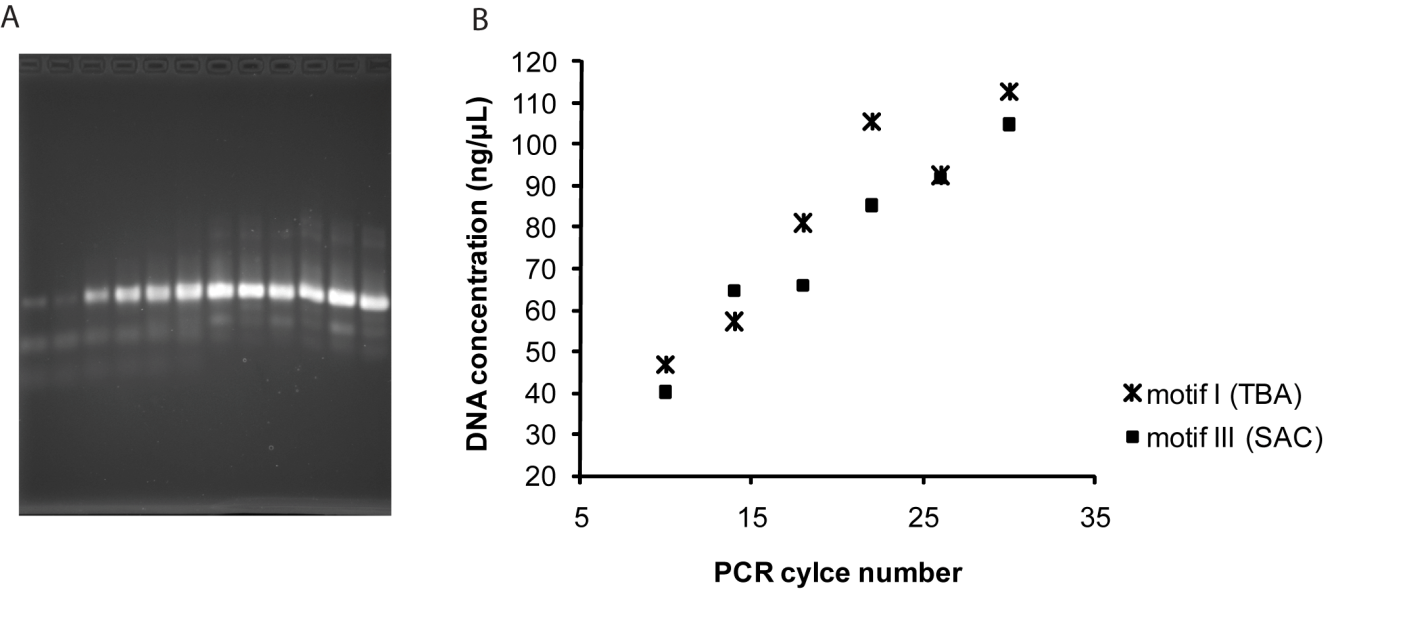
**

**Figure S2. Investigation of potential amplification bias in aptamer candidates by semi-quantitative real time PCR.** **A, B** Analysis of PCR-amplification rates by gel electrophoresis and absorbance measurements at 260nm. In panel **A** are six pairs of PCR time points for Thb1 (motif I) and Carb1 (motif II), respectively, in a 2% agarose gel (cycle numbers: 10, 14, 18, 22, 26, 30) and in **B,** are the 260nm readings of a duplicate experiment.
