# Supplementary material for: Acyclic Identification of Aptamers for Human alpha-Thrombin Using Over-Represented Libraries and Deep Sequencing
Source: PLoS One. 2011 May 19;6(5):e19395. doi: 10.1371/journal.pone.0019395 (PMC3098231; doi:10.1371/journal.pone.0019395)
Supplement: Figure S3 — Background determination in thrombin aptamer identification. Following deep sequencing, the occurrence of each sequence was determined and ranked. High occurrence sequences were aligned to determine conserved motifs. The panels show phylogenetic trees generated from sequences counted 10 times or higher, 9+, 8+, 7+, 6+, 5+ and about one thousand of 4+, respectively. Replicate experiments consistently had large numbers of uncorrelated sequences, starting at a count of 4 to 6. (DOCX) [file pone.0019395.s003.docx]

**
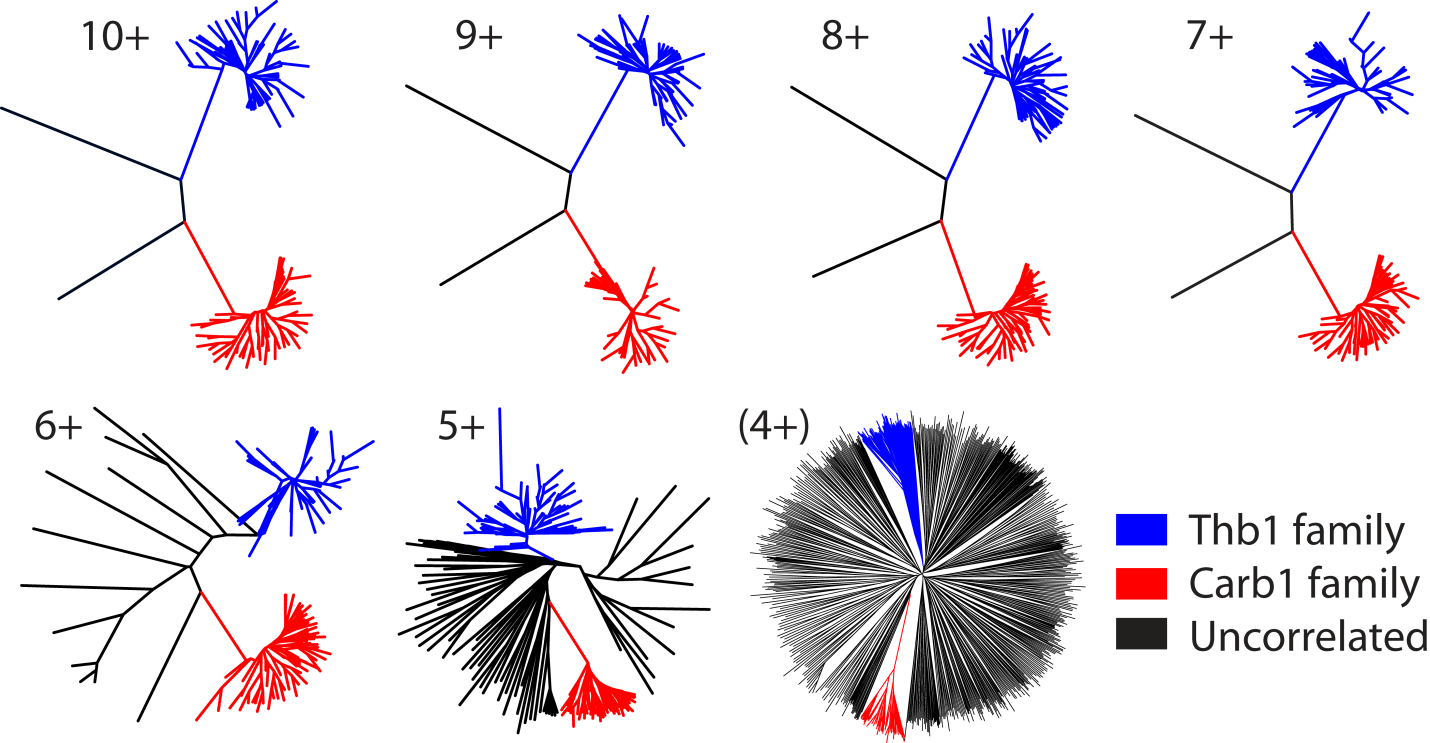
**

**Figure S3. Background determination in thrombin aptamer identification.** Following deep sequencing, the occurrence of each sequence was determined and ranked. High occurrence sequences were aligned to determine conserved motifs. The panels show phylogenetic trees generated from sequences counted 10 times or higher, 9+, 8+, 7+, 6+, 5+ and about one thousand of 4+, respectively. Replicate experiments consistently had large numbers of uncorrelated sequences, starting at a count of 4 to 6.
