# Supplementary material for: Acyclic Identification of Aptamers for Human alpha-Thrombin Using Over-Represented Libraries and Deep Sequencing
Source: PLoS One. 2011 May 19;6(5):e19395. doi: 10.1371/journal.pone.0019395 (PMC3098231; doi:10.1371/journal.pone.0019395)
Supplement: Figure S4 — Systematic artifacts in surviving sequences. The full adapter-ligated construct prior to PCR-amplification is shown in A. Highlighted in yellow are the stem and tails of the hairpin library designed for the 15mer thrombin experiment, while highlighted in green, the letters, N, signify the 15mer library loop region. The red bases are the flanking regions used by the Perl script to find qualifying reads. In the case of the most abundant systematic artifact, the Perl script recognized the 5′ flanking region but substituted the second flanking region with the adapter sequence underlined above, thereby presenting the first 15 bases of adapter 2 as the library region. This could only occur with the jump shown in dotted line and from the sequence shown in B. (DOCX) [file pone.0019395.s004.docx]

**Table S4. Results from three replicate selection and identification experiments for α-thrombin with increasing hexose sugar content.**^a^

|  | **Experiment T1** | | **Experiment T2** | | **Experiment T3** | |
| --- | --- | --- | --- | --- | --- | --- |
| **Rank** | **m=15 loop** | **Count**^b^ | **m=15 loop** | **Count**^c^ | **m=15 loop** | **Count**^d^ |
| **1** | GGTTGGTGTGGTTGG | 46,444 | **GcTaTcaTcGcaacG** | 39,940 | **GcTaTcaTcGcaacG** | 36,636 |
| **2** | **GcTaTcaTcGcaacG** | 29,405 | GGTTGGTGTGGTTGG | 24,484 | caTGcGccacaGaTc | 7,435 |
| **3** | GGTTGGTGTGGTTTG | 2,451 | GcTaTcaTcGcaccG | 2,038 | GGTTGGTGTGGTTGG | 5,140 |
| **4** | GcTaTcaTcGccacG | 1,040 | GcTaTcaTcGccacG | 1,404 | aGaTcGGaaGaGcTc | 4,848 |
| **5** | aGaTcGGaaGaGcTc | 710 | GGTTGGTGTGGTTTG | 1,164 | aTGcGccacaGaTcG | 494 |
| **6** | GcTaTcaTcGcaccG | 678 | GGTTGGTGTGGTTGT | 802 | GaTcGGaaGaGcaTc | 247 |
| **7** | GGTTGGTGTTGTTGG | 647 | GGTGGTTGTTGGTGT | 652 | ccacaGaTcGGaaGa | 191 |
| **8** | GGTTGGTGTGGTTGT | 591 | GcTcTcaTcGcaacG | 432 | GcacaGaTcGGaaGa | 146 |
| **9** | GGTTGGTTTGGTTGG | 419 | GGGTGGTGTGGTTGG | 380 | aGaGcGGaaGaGcTc | 106 |
| **10** | GcTcTcaTcGcaacG | 354 | GcTaTcaTcGcaacT | 282 | GcTaGcaTcGcaacG | 103 |
| **11** | GGcTGGTGTGGTTGG | 255 | GcTaTcaTcTcaacG | 271 | GcGaTcaTcGcaacG | 103 |
| **12** | GcTaTcaTcGcaacc | 220 | GGcTGGTGTGGTTGG | 253 | GcaTGcGcacaGaTc | 89 |
| **13** | GGTTGGTGTGTTTGG | 215 | GccacaGaTcGGaaG | 247 | aTcGGaaGaGcGTcG | 86 |
| **14** | GcTaTccTcGcaacG | 199 | GGTTGGGGTGGTTGG | 243 | GcTaTcaTcGcGacG | 86 |
| **15** | GGTTGGcGTGGTTGG | 195 | GcTaTcaTcccaacG | 228 | GcTaTcaTcGcaaaG | 84 |

a. Results from three experiments are shown in order of increasing (left to right) concentration of hexose sugars (glucose and alpha methyl mannoside). Underlined sequences are related to Carb1, which is shown in bold. The ratio of counts for Carb1/Thb1 is 0.6, 1.6, and 7.1, respectively for the three experiments.

b. A total of 1,959,748 reads had validated stems separated by 15 bases, with 1,728,220 unique sequences found.

c. A total of 4,507,157 reads had validated stems separated by 15 bases, with 4,106,253 unique sequences found.

d. A total of 1,470,764 reads had validated stems separated by 15 bases, with 1,393,127 unique sequences found.
