# Supplementary material for: Acyclic Identification of Aptamers for Human alpha-Thrombin Using Over-Represented Libraries and Deep Sequencing
Source: PLoS One. 2011 May 19;6(5):e19395. doi: 10.1371/journal.pone.0019395 (PMC3098231; doi:10.1371/journal.pone.0019395)
Supplement: Figure S5 — Thrombin analysis by MALDI TOF mass spectrometry. Purity of α-thrombin was verified by MALDI TOF mass spectrometry prior to use in selection experiments and was consistently >90% pure with minimal degradation. (DOCX) [file pone.0019395.s005.docx]

**
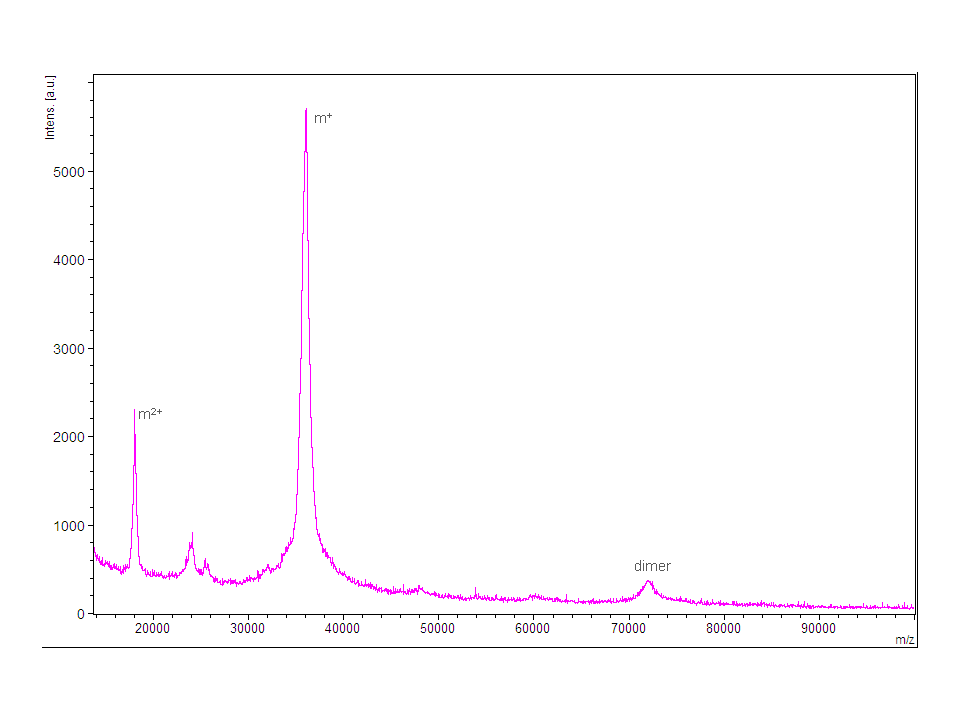
**

**Figure S5. Thrombin analysis by MALDI TOF mass spectrometry.** Purity of α-thrombin was verified by MALDI TOF mass spectrometry prior to use in selection experiments and was consistently >90% pure with minimal degradation.
