# Supplementary material for: Acyclic Identification of Aptamers for Human alpha-Thrombin Using Over-Represented Libraries and Deep Sequencing
Source: PLoS One. 2011 May 19;6(5):e19395. doi: 10.1371/journal.pone.0019395 (PMC3098231; doi:10.1371/journal.pone.0019395)
Supplement: Figure S6 — Sample preparation for high throughput sequencing after selection. A. Confirmation of the ∼120 base pair (bp) ligation product on a 2% agarose gel. The ligation product was excised from the gel, purified and PCR-amplified. B. Size confirmation of the final selected, ligated and PCR-amplified pool prior to DNA sequencing. In the first lane of both pictures is a 50 bp DNA ladder. In lane 2 of A is the ligation product before excision and clean up. In lanes 2 and 3 of B are a negative PCR-control and the PCR-product respectively. (DOCX) [file pone.0019395.s006.docx]

**
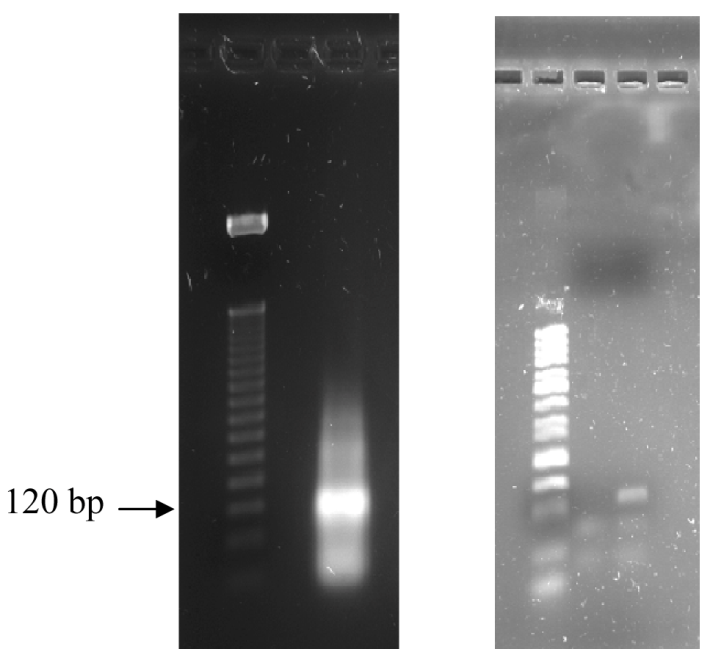
**

**A B**

**Figure S6. Sample preparation for high throughput sequencing after selection.** **A.** Confirmation of the ~120 base pair (bp) ligation product on a 2% agarose gel. The ligation product was excised from the gel, purified and PCR-amplified. **B.** Size confirmation of the final selected, ligated and PCR-amplified pool prior to DNA sequencing. In the first lane of both pictures is a 50 bp DNA ladder. In lane 2 of **A** is the ligation product before excision and clean up. In lanes 2 and 3 of **B** are a negative PCR-control and the PCR-product respectively.
