# Supplementary material for: Acyclic Identification of Aptamers for Human alpha-Thrombin Using Over-Represented Libraries and Deep Sequencing
Source: PLoS One. 2011 May 19;6(5):e19395. doi: 10.1371/journal.pone.0019395 (PMC3098231; doi:10.1371/journal.pone.0019395)
Supplement: Table S1 — Representation of sequences in naïve pools. (DOCX) [file pone.0019395.s007.docx]

**Table S1. Representation of sequences in naïve pools.**

| **m** | **U** | **R(0.1)** | **R(25)** |
| --- | --- | --- | --- |
| **1** | 4.0E+00 | 1.5E+13 | 3.8E+15 |
| **2** | 1.6E+01 | 3.8E+12 | 9.4E+14 |
| **3** | 6.4E+01 | 9.4E+11 | 2.4E+14 |
| **4** | 2.6E+02 | 2.4E+11 | 5.9E+13 |
| **5** | 1.0E+03 | 5.9E+10 | 1.5E+13 |
| **6** | 4.1E+03 | 1.5E+10 | 3.7E+12 |
| **7** | 1.6E+04 | 3.7E+09 | 9.2E+11 |
| **8** | 6.6E+04 | 9.2E+08 | 2.3E+11 |
| **9** | 2.6E+05 | 2.3E+08 | 5.7E+10 |
| **10** | 1.0E+06 | 5.7E+07 | 1.4E+10 |
| **11** | 4.2E+06 | 1.4E+07 | 3.6E+09 |
| **12** | 1.7E+07 | 3.6E+06 | 9.0E+08 |
| **13** | 6.7E+07 | 9.0E+05 | 2.2E+08 |
| **14** | 2.7E+08 | 2.2E+05 | 5.6E+07 |
| **15** | 1.1E+09 | 5.6E+04 | 1.4E+07 |
| **16** | 4.3E+09 | 1.4E+04 | 3.5E+06 |
| **17** | 1.7E+10 | 3.5E+03 | 8.8E+05 |
| **18** | 6.9E+10 | 8.8E+02 | 2.2E+05 |
| **19** | 2.7E+11 | 2.2E+02 | 5.5E+04 |
| **20** | 1.1E+12 | 5.5E+01 | 1.4E+04 |
| **21** | 4.4E+12 | 1.4E+01 | 3.4E+03 |
| **22** | 1.8E+13 | 3.4E+00 | 8.6E+02 |
| **23** | 7.0E+13 | 8.6E-01 | 2.1E+02 |
| **24** | 2.8E+14 | 2.1E-01 | 5.3E+01 |
| **25** | 1.1E+15 | 5.3E-02 | 1.3E+01 |
| **26** | 4.5E+15 | 1.3E-02 | 3.3E+00 |
| **27** | 1.8E+16 | 3.3E-03 | 8.4E-01 |
| **28** | 7.2E+16 | 8.4E-04 | 2.1E-01 |
| **29** | 2.9E+17 | 2.1E-04 | 5.2E-02 |
| **30** | 1.2E+18 | 5.2E-05 | 1.3E-02 |
| **35** | 1.2E+21 | 5.1E-08 | 1.3E-05 |
| **40** | 1.2E+24 | 5.0E-11 | 1.2E-08 |
| **60** | 1.3E+36 | 4.5E-23 | 1.1E-20 |
| **80** | 1.5E+48 | 4.1E-35 | 1.0E-32 |

1. m = length of randomized sequence.
2. U = 4^m^, the number of possible unique randomized sequences of length, m. Pools with U > 3E+17 are beyond the synthesizer limit for a 1 μmol scale synthesis (99% stepwise coupling efficiency with 32 nt fixed-sequence regions).
3. R = A/U, the average representation of each unique molecule in a pool; A = the number of all strands in the pool (6E+13 for 0.1 nmol and 1.5E+16 for 25 nmol of the pool). Large pools with R < 3 are beyond the diversity limit. To achieve R = 3, about 1,400 kg of DNA with m = 40 would be required, and a mass of DNA about 450 times that of the earth for m = 80.
