# Supplementary material for: Acyclic Identification of Aptamers for Human alpha-Thrombin Using Over-Represented Libraries and Deep Sequencing
Source: PLoS One. 2011 May 19;6(5):e19395. doi: 10.1371/journal.pone.0019395 (PMC3098231; doi:10.1371/journal.pone.0019395)
Supplement: Table S2 — Files generated by Perl script. (DOCX) [file pone.0019395.s008.docx]

**Table S2. Files generated by Perl script.**

| **File** | **Description of Contents** |
| --- | --- |
| **Location Statistics** | NN**NNNNNNNNNN**m**NNNNN**NNNNNNN  Counts of each base in constant regions in the 5′- and 3′-flanking regions (F5 and F3) for each sequence in the text file generated by the Illumina Genome Analyzer. These are compared to the expected sequence to determine errors ascribed to PCR and/or sequencing. |
| **Qualified reads** | NNNNNNNNNNNN m = target m NNNNNNNNNNNN  Sequences with satisfactory F5 and F3 that also have exactly 15 bases in the library region. F5 barcodes of ten bases were allowed two and F3 barcodes of five bases allowed one substitution, deletion, or insertion. |
| **Candidate reads** | NNNNNNNNNNNN m ≠ target m NNNNNNNNNNNN  Sequences that possess the satisfactory barcodes in F5 and F3 but have the wrong oligonucleotide length in the randomized region. These may be due to insertions and deletions in the randomized region. |
| **Bad reads** | NN**NNNNNNNNNN**m**NNNNN**NNNNNNN  Sequences that possessed unacceptable F5 or F3 sequences. These include DNA contaminants or heavily mutated or misread sequences. |
| **Nmer count** | Counts and ranking of qualified reads. |

**Notes:**

N = constant region DNA not used in analysis, **N** = unsorted sequence from parent sequencing file, **N** = incorrect sequence in F5 or F3, N = correct stem base, m = library region
