# Supplementary material for: Acyclic Identification of Aptamers for Human alpha-Thrombin Using Over-Represented Libraries and Deep Sequencing
Source: PLoS One. 2011 May 19;6(5):e19395. doi: 10.1371/journal.pone.0019395 (PMC3098231; doi:10.1371/journal.pone.0019395)
Supplement: Table S3 — Base calling statistics for the fixed-sequences flanking the m = 15 library region. (DOCX) [file pone.0019395.s009.docx]

**Table S3. Base calling statistics for the fixed-sequences flanking the m = 15 library region.**^a^

|  | **Match** | **Mismatch** | **Deletion** | **Insertion** |
| --- | --- | --- | --- | --- |
| **F5 (10 bases analyzed)**^b^ | | | | |
| **A3** | 99.85 | 0.15 | 0.00 | 0.00 |
| **C4** | 99.93 | 0.07 | 0.00 | 0.00 |
| **G5** | 99.81 | 0.12 | 0.08 | 0.08 |
| **C6** | 99.89 | 0.07 | 0.04 | 0.09 |
| **G7** | 99.86 | 0.08 | 0.06 | 0.04 |
| **C8** | 99.88 | 0.09 | 0.03 | 0.07 |
| **A9** | 99.74 | 0.15 | 0.11 | 0.04 |
| **T10** | 99.82 | 0.10 | 0.08 | 0.12 |
| **G11** | 99.78 | 0.14 | 0.09 | 0.08 |
| **C12** | 99.81 | 0.19 | 0.00 | 0.09 |
| **F3 (5 bases analyzed)**^c^ | | | | |
| **G28** | 97.89 | 2.11 | 0.00 | 0.00 |
| **C29** | 99.06 | 0.94 | 0.00 | 0.00 |
| **A30** | 95.52 | 4.48 | 0.00 | 0.01 |
| **T31** | 99.78 | 0.22 | 0.00 | 0.01 |
| **G32** | 95.29 | 4.71 | 0.00 | 0.00 |

1. Analysis of qualified stems from experiment T1; the percentages for perfect matches, mismatches, deletions, and insertions are shown.
2. Bases C1 and A2 in F5 were read using the Illumina GA but not used in the Perl script..
3. Bases 13-27 comprise the m = 15 library region. This is followed by F3 (G28-A39), of which G28-G32 were analyzed using the Perl script.
