# Supplementary material for: Acyclic Identification of Aptamers for Human alpha-Thrombin Using Over-Represented Libraries and Deep Sequencing
Source: PLoS One. 2011 May 19;6(5):e19395. doi: 10.1371/journal.pone.0019395 (PMC3098231; doi:10.1371/journal.pone.0019395)
Supplement: Table S5 — Statistics for the sequence space encompassing the Thb1 motif. (DOCX) [file pone.0019395.s011.docx]

**Table S5. Statistics for the sequence space encompassing the Thb1 motif.**^a^

| **Position** | **G** | **C** | **A** | **T** |
| --- | --- | --- | --- | --- |
| **G1** | **99.69** | 0.13 | 0.09 | 0.09 |
| **G2** | **99.77** | 0.12 | 0.07 | 0.04 |
| **T3** | 0.33 | 0.62 | 0.05 | **99.00** |
| **T4** | 0.06 | 0.18 | 0.01 | **99.75** |
| **G5** | **99.43** | 0.17 | 0.05 | 0.35 |
| **G6** | **99.52** | 0.13 | 0.04 | 0.31 |
| **T7** | 0.33 | 0.46 | 0.08 | **99.12** |
| **G8** | **98.62** | 0.18 | 0.06 | 1.14 |
| **T9** | 0.16 | 0.23 | 0.05 | **99.57** |
| **G10** | **97.94** | 0.18 | 0.05 | 1.82 |
| **G11** | **99.22** | 0.11 | 0.04 | 0.63 |
| **T12** | 0.22 | 0.30 | 0.04 | **99.45** |
| **T13** | 0.04 | 0.21 | 0.03 | **99.72** |
| **G14** | **94.19** | 0.15 | 0.04 | 5.62 |
| **G15** | **98.17** | 0.19 | 0.03 | 1.61 |

1. The frequency of occurrence for each base is shown for the m = 15 randomized positions, given as a percentage of the top 54,140 counts comprising the top 108 sequences of the Thb1 motif.
